# Strain-induced perpendicular magnetic anisotropy and Gilbert damping of Tm_3_Fe_5_O_12_ thin films

**DOI:** 10.1038/s41598-019-53255-6

**Published:** 2019-11-25

**Authors:** Oana Ciubotariu, Anna Semisalova, Kilian Lenz, Manfred Albrecht

**Affiliations:** 1 Institute of Physics, University of Augsburg, Universitätsstraße 1, 86135 Augsburg, Germany; 2 Institute of Ion Beam Physics and Materials Research, Helmholtz-Zentrum Dresden-Rossendorf, Bautzner Landstraße 400, 01328 Dresden, Germany; 3 Present Address: Faculty of Physics, University of Duisburg-Essen, Lotharstraße 1, 47057 Duisburg, Germany

**Keywords:** Magnetic properties and materials, Spintronics

## Abstract

In the attempt of implementing iron garnets with perpendicular magnetic anisotropy (PMA) in spintronics, the attention turned towards strain-grown iron garnets. One candidate is Tm_3_Fe_5_O_12_ (TmIG) which possesses an out-of-plane magnetic easy axis when grown under tensile strain. In this study, the effect of film thickness on the structural and magnetic properties of TmIG films including magnetic anisotropy, saturation magnetization, and Gilbert damping is investigated. TmIG films with thicknesses between 20 and 300 nm are epitaxially grown by pulsed laser deposition on substituted-Gd_3_Ga_5_O_12_(111) substrates. Structural characterization shows that films thinner than 200 nm show in-plane tensile strain, thus exhibiting PMA due to strain-induced magnetoelastic anisotropy. However, with increasing film thickness a relaxation of the unit cell is observed resulting in the rotation of the magnetic easy axis towards the sample plane due to the dominant shape anisotropy. Furthermore, the Gilbert damping parameter is found to be in the range of 0.02 ± 0.005.

## Introduction

Yttrium iron garnet (Y_3_Fe_5_O_12_, YIG) is an insulating ferrimagnet with an extremely low Gilbert damping parameter in the order of 10^−4^ ^1,2^ making it the ideal choice for spin transport studies, including inverse spin Hall effect (ISHE)^3^, spin Seebeck effect^4^, spin wave propagation^5^, and spin colossal magnetoresistance^6^. YIG layers exhibiting a cubic structure (space group Ia-3d) with a lattice parameter of *a* = 12.376 Å are commonly grown on Gd_3_Ga_5_O_12_ (GGG, *a* = 12.383 Å) substrates due to the small lattice mismatch (∆ = 100(*a*_sub_ − *a*_film_)/*a*_sub_ = 0.05%) by pulsed laser deposition (PLD)^1–6^ or sputtering^7^. In thin film form, the magnetic shape anisotropy overcomes the weak magnetocrystalline anisotropy^8^, thus revealing an in-plane (ip) magnetic easy axis. Nevertheless, tailoring the magnetic easy axis direction would open new potential applications in the field of magnon spintronics^9^. One method for achieving perpendicular magnetic anisotropy (PMA) is by induced strain giving rise to magnetoelastic anisotropy. According to refs. ^10,11^, iron garnets with a negative (positive) magnetostriction constant (*λ*_111_) can have an out-of-plane (oop) easy axis of magnetization when grown under in-plane tensile (compressive) strain. Several approaches have been proposed to achieve for YIG layers an oop easy axis of magnetization, including the variation of oxygen pressure during growth and substrate type^12^. However, in these studies no PMA could be observed for YIG films grown on GGG(111) and substituted-GGG (sGGG, *a* = 12.505 Å) (111) substrates. In contrast, Krockenberger *et al*. observed for 100 nm thick YIG films grown on GGG(111) substrates weak PMA associated with a slight elongation of the unit cell along the surface normal^13^. Additionally, Popova *et al*. reported for 10 nm thick polycrystalline YIG films deposited on quartz substrates by PLD the presence of weak PMA with low remanent magnetization^14,15^. Recently, tensile strain-induced magnetoelastic anisotropy was observed for YIG films grown on (Gd_0.63_Y_2.37_)(Sc_2_Ga_3_)O_12_ (∆ = 1.04%) and Gd_3_(Sc_2_Ga_3_)O_12_ (∆ = 1.56%) substrates leading to an oop magnetic easy axis for film thicknesses below 15 nm^16^. Additionally, stress-engineered 20–40 nm thick YIG films sandwiched between a buffer and a cover layer revealed a suppressed in-plane strain relaxation, thus, allowing the presence of PMA in the order of 12 kJ/m^3^ ^17^. Furthermore, adding third elements can induce sufficient in-plane tensile strain to the unit cell resulting in strain-induced PMA as demonstrated for Bi-doped YIG films on sGGG(111) with little impact on the Gilbert damping parameter (*α* = 3 × 10^−4^)^18^.

In the attempt of improving PMA for larger film thicknesses, the attention turned to other rare earth-based iron garnets while trying to maintain a low Gilbert damping parameter^12,19,20^. A promising candidate is Tm_3_Fe_5_O_12_ (TmIG, *a* = 12.323 Å). Due to the negative magnetostriction constant of *λ*_111_ = −5.2 × 10^−6^ ^8^, possible substrate choices for the growth of TmIG films under tensile strain are GGG (∆ = 0.49%) and sGGG (∆ = 1.45%) substrates. Since for most garnets the [111] direction is the crystallographic easy axis of magnetization^8^, (111)-oriented substrates are commonly chosen. Tang *et al*. observed for 10 nm thick TmIG films an oop easy axis of magnetization when grown on sGGG(111) substrates while 10 nm thick TmIG layers on GGG(111) substrates showed an ip easy axis of magnetization, suggesting an insufficient tensile strain for the latter case^21^. A similar result was recently found by Ahmed *et al*. for extremely thin layers (<2 nm)^22^. However, Kubota *et al*. reported the growth of epitaxial TmIG films (46–350 nm) on GGG(111) by PLD under tensile strain revealing PMA^19^. Films thinner than 30 nm were observed to have similar magnetic properties with a magnetoelastic anisotropy of 17.5 kJ/m^3^ ^23,24^. In a recent study, off-axis sputter-deposited TmIG films on GGG(111) in the thickness range of 10–30 nm showed PMA and low Gilbert damping of *α* = 0.0133^25^, still being two orders of magnitude higher than for YIG films. Hence, subtle differences regarding growth and strain conditions obviously influence the overall magnetic properties of rare earth-based iron garnet films.

The presented study is focusing on the evolution of the structural and magnetic properties of TmIG thin films with film thickness. Films were grown epitaxially by PLD at 615 °C on sGGG(111), allowing for a higher in-plane tensile strain than GGG(111) substrates. Furthermore, the Gilbert damping parameter was determined.

## Methods

Tm_3_Fe_5_O_12_ films were grown by PLD from a commercially available polycrystalline Tm_3_Fe_5_O_12_ target (G-Materials GmbH) with a purity of 99.99%. Prior to deposition, the target was cleaned under vacuum (10^−6^ mbar) with 10,000 laser pulses at an energy of 300 mJ (pulse length: 20 ns, wavelength: 248 nm). In the PLD chamber, the substrate was placed approximately 4 cm in front of the target and the temperature was monitored via a thermocouple placed in the sample holder. For a good thermal contact with the sample holder, the back side and the edges of the substrates were covered with silver paste.

In order to identify the growth conditions of stoichiometric films, TmIG films (110–160 nm) were prepared on silicon substrates at 650 °C while the oxygen pressure (*p*O_2_) was varied between 0.04 and 0.4 mbar. The films stoichiometry and the deposition rates were analyzed by Rutherford backscattering spectrometry (RBS) using the SIMNRA program^26^. For this study, we have chosen silicon instead of sGGG substrates in order to extract reliable values for the composition and thickness due to the small atomic mass difference between Fe and Ga, and Tm and Gd atoms resulting in an overlap of the corresponding peaks in the RBS spectra. All spectra were acquired for an incident He^2^^+^ ion energy of 3.0 MeV. The RBS analysis showed that for *p*O_2_ = 0.04 mbar the Tm/Fe ratio is close to the stoichiometric value of 0.6. With increasing *p*O_2_, the films become Tm-rich and for *p*O_2_ = 0.4 mbar a Tm/Fe ratio as high as 0.7 was measured. Please note that the reported values for the oxygen pressure typically used for TmIG growth are in a higher range of 0.26–1.06 mbar^19,23,24^, but this depends strongly on target stoichiometry. Further studies on TmIG(70–100 nm)/sGGG(111) regarding substrate temperature, laser pulse energy, and pulse rate using an oxygen pressure of 0.04 mbar revealed an optimum at 615 °C, 300 mJ, and 2 Hz, respectively. Under these conditions stoichiometric TmIG films with thicknesses between 20 and 300 nm were grown epitaxially by PLD on sGGG(111) substrates (at a rate of 0.03 nm/s). After deposition, the samples were cooled with a rate of −13 °C/min although in literature smaller values are reported for iron garnets^24,27^. The impact of the cooling rate was investigated on 70 nm thick TmIG films cooled with different rates between −2 °C/min and −13 °C/min. However, only a minor impact of the cooling rate on the structural and magnetic properties was observed (not shown here).

The surface morphology of the films was investigated by atomic force microscopy (AFM) and the structural characterization was carried out by x-ray diffraction (XRD) whereas the magnetic properties were investigated by superconducting quantum interference device – vibrating sample magnetometry (SQUID–VSM). Before the magnetic characterization, the sample edges were cut using a diamond saw and the backside was polished using sand paper in order to remove the silver paste. Furthermore, a correction of the magnetic field values was required due to the remanent magnetic field (approximately 30 Oe) of the superconducting coils. For this procedure a paramagnetic palladium reference sample was measured following the same sequence as the one carried out for the TmIG samples. For a paramagnetic sample, the field dependence of the magnetization is a straight line passing through zero. As a result of the trapped magnetic field in the superconducting material, an open hysteresis loop is observed for the reference sample. By applying a fitting routine for correcting the magnetic field values, a straight line passing through zero is restored. Ferromagnetic resonance (FMR) measurements were performed to extract the magnetic anisotropy and the damping parameter using a Vector Network Analyzer (VNA) setup. An Agilent VNA was used to excite the ferromagnetic resonance in the thin film placed on a coplanar waveguide in the range of 1–40 GHz. The complex microwave scattering parameter *S*_21_, i.e. the transmission signal between the VNA ports, was recorded as the FMR signal while the external magnetic field was swept at a fixed microwave frequency.

## Results and Discussions

**Figure 1:**
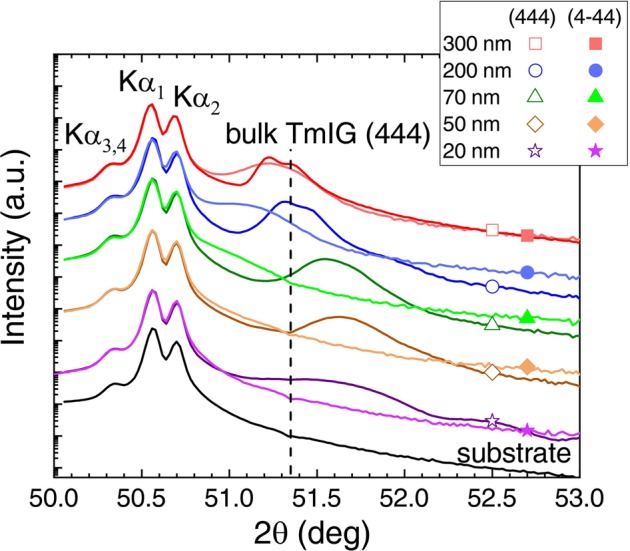
XRD *θ*–2*θ* scans of the (444) and (4–44) planes of TmIG/sGGG(111) samples for various film thicknesses and of a bare substrate. The position of bulk TmIG (444) reflection is marked with a vertical line.

Figure 1 shows XRD *θ*–2*θ* scans measured for all samples around the sGGG (444) reflection. The diffraction pattern of a bare substrate is displayed for comparison, revealing a splitting of the main (444) reflection due to contributions from the Cu Kα_1_, Cu Kα_2_, and Cu Kα_3,4_ radiations^28,29^, which are sometimes misinterpreted in the literature^30^.

From the XRD patterns, a strong influence of the TmIG film thickness on the crystalline structure is apparent. All films with thicknesses below 200 nm have the (444) diffraction peak at 2*θ* angles larger than for bulk TmIG^31^, which indicates a compressed lattice in growth direction. This compression should result in an expanded lattice in the film plane inducing tensile strain, the required condition for obtaining an out-of-plane magnetoelastic anisotropy contribution. The largest distortion is measured for the thinnest films of 20 and 50 nm. With increasing film thickness, the (444) peak shifts towards smaller angles indicating a structural relaxation towards the bulk structure. Please note that a slight off-stoichiometry can explain the difference between the (444) peak position of bulk and 300 nm thick film.

For a cubic system (e.g. sGGG and TmIG), the <111> directions are equivalent and, according to Bragg’s law, the corresponding diffraction planes have the same diffraction angle. Therefore, the distortion of the unit cell was investigated from the position of the (4–44) reflection. To access the (4–44) diffraction plane, each sample was tilted to *χ* = 70.53°. The corresponding scans are included in Fig. 1. For the sGGG substrate, due to the cubic crystal structure, the (444) and (4–44) reflections overlap perfectly. In contrast, pronounced differences are visible between the (444) and (4–44) reflections of the films clearly confirming the strong lattice distortion. For the 20 and 50 nm thick films, the (4–44) diffraction peak is hardly visible as a shoulder on the right side of the substrate peak. With increasing film thickness, the (4–44) diffraction peak shifts towards higher angles approaching the (444) reflection which indicates the relaxation into the cubic structure.

**Figure 2:**
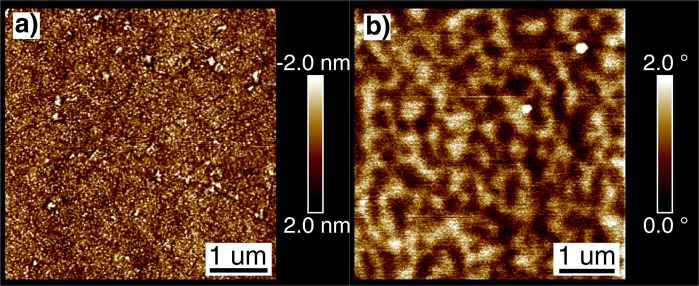
(**a**) Surface topography and (**b**) magnetic domain structure of a 70 nm thick TmIG film grown on sGGG(111). Please note that the images were taken at different sample areas.

The surface morphology of the 70 nm thick TmIG film was investigated by AFM showing a root mean square (rms) roughness of about 0.8 nm extracted from the topographic image presented in Fig. 2(a). Throughout the series, all film samples exhibit a smooth and continuous surface with a rather low rms roughness of less than 1 nm.

**Figure 3:**
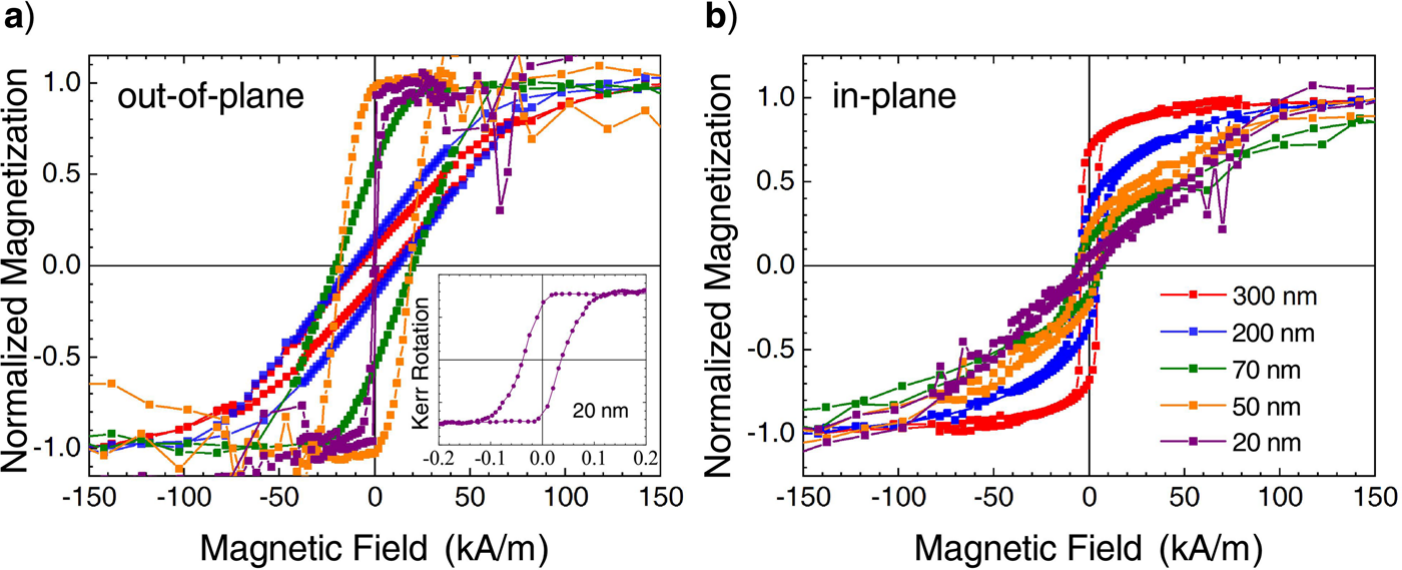
Normalized *M*–*H* hysteresis loops measured in (**a**) out-of-plane and (**b**) in-plane geometry. The paramagnetic substrate contribution was subtracted from the measured data. Inset displays the polar MOKE loop measured for the 20 nm thick film.

It is expected that the observed structural changes will strongly influence the magnetic properties. In order to possess an out-of-plane magnetic easy axis, the induced magnetoelastic anisotropy has to overcome the magnetic shape anisotropy of the film (the magnetocrystalline contribution is discussed below). Figure 3 displays the normalized *M*–*H* hysteresis loops measured with the magnetic field applied in the oop and ip direction after subtraction of the substrate paramagnetic contribution.

The 50 nm thick TmIG film exhibits a rather square hysteresis loop in oop direction with a coercivity of about 18.3 kA/m (230 Oe). For the ip direction, a small hysteresis with a lower coercivity of 4.7 kA/m (60 Oe) and a linearly increasing magnetization towards saturation is obtained, confirming PMA with an easy axis along the surface normal. Thus, for this film thickness, the magnetoelastic anisotropy is dominant. A similar behavior is expected for the 20 nm thick TmIG film. However, in case of weak magnetic film signals, the hysteresis loops become noisy, in particular at higher fields, after removing the substrate contribution (see Fig. 3 for 20 and 50 nm thick films). Therefore, an additional polar magneto-optical Kerr effect (MOKE) hysteresis loop was measured using a blue laser source (*λ* = 405 nm) and it is displayed in the inset of Fig. 3(a). The MOKE loop revealed a similar shape as observed by SQUID–VSM and confirmed the oop easy axis of magnetization with a rather low coercivity of about 2.95 kA/m (37 Oe). With increasing film thickness, the magnetic easy axis rotates towards the sample plane due to the loss in magnetoelastic anisotropy and competing contribution of the magnetic shape anisotropy.

**Figure 4:**
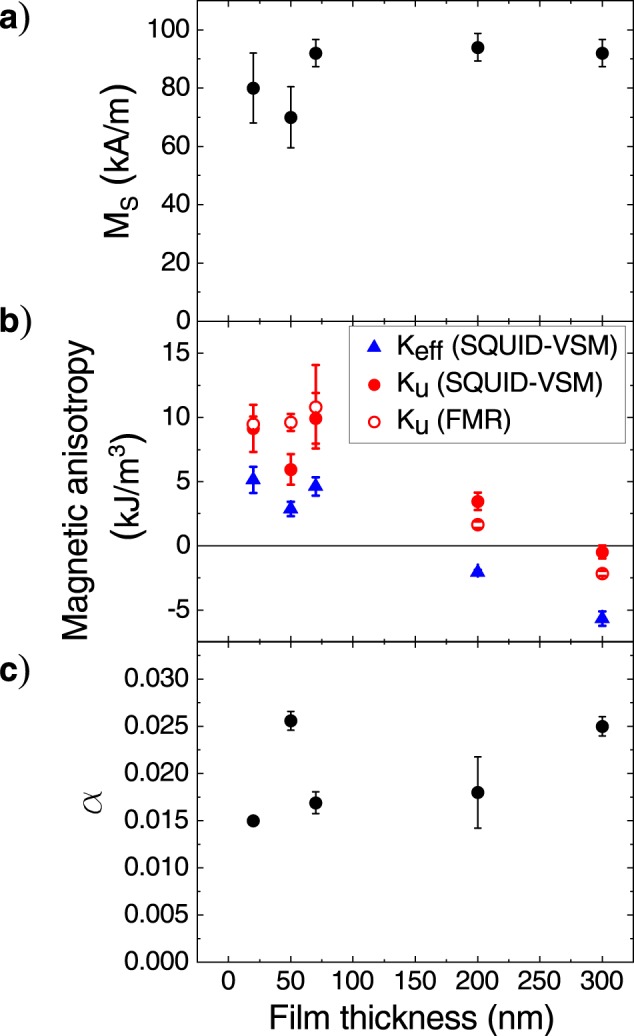
Dependence of (**a**) saturation magnetization, (**b**) magnetic anisotropy, and (**c**) Gilbert damping parameter on TmIG film thickness.

The saturation magnetization (*M*_S_) in dependence on the TmIG film thickness is shown in Fig. 4(a). The obtained *M*_S_ values of about 90 kA/m (90 emu/cm^3^) remain almost constant within the experimental error, which is smaller than for bulk TmIG (110 kA/m) but in good agreement with values reported by other groups^23–25,32^.

The values of the effective magnetic anisotropy *K*_eff_ are summarized in Fig. 4(b). *K*_eff_ was estimated from the area enclosed between the out-of-plane and in-plane loops in one quadrant of the *M*−*H* loops and contains both perpendicular *K*_u_ and shape *K*_s_ (*K*_s_ = −1/2μ_0_*M*_S_^2^) anisotropies.

With the values of *K*_eff_ and *M*_S,_ the perpendicular magnetic anisotropy energy density *K*_u_ = *K*_eff_ − *K*_s_ (filled red circles in Fig. 4(b)) was calculated to be around 6–10 kJ/m^3^ (60–100 kerg/cm^3^) for films thicknesses up to 70 nm. With increasing film thickness, the *K*_u_ values get reduced and eventually the magnetization rotates in the film plane as indicated by the sign change of *K*_eff_.

As mentioned above, <111> are the crystallographic easy axes. Therefore, the two contributions to the perpendicular magnetic anisotropy *K*_u_ (also often denoted as *K*_2⊥_) are the magnetocrystalline anisotropy *K*_1_ and the magnetoelastic anisotropy *K*_σ_ induced by tensile strain. For bulk TmIG, the first order cubic anisotropy constant *K*_1_ (often denoted as *K*_4_) is in the range of −1.1 kJ/m^3^ < *K*_1_ < −0.58 kJ/m^3^ and it is often neglected^8^. Based on ref. ^33^, the strain-induced magnetoelastic anisotropy *K*_σ_ can be estimated using the formula

$${K}_{{\rm{\sigma }}}=-\frac{3}{2}\frac{Y}{1+\mu }{\lambda }_{111}\frac{{d}_{{\rm{bulk}}}^{444}-{d}_{{\rm{film}}}^{444}}{{d}_{{\rm{bulk}}}^{444}}$$

where *λ*_111_ = −5.2×10^−6^ is the magnetostriction constant in the [111] direction. Instead of the Young’s modulus *Y* and Poisson ratio *μ* of TmIG, the values of *Y* = 2×10^8^ kJ/m^3^ and *μ* = 0.29 of YIG were used^33^. The film interplanar distance ${d}_{{\rm{film}}}^{444}$ was calculated using Bragg’s law and the film diffraction angle extracted from fittings of the XRD patterns. Considering that the TmIG films converge towards an oop lattice constant larger than of stoichiometric bulk TmIG (see Fig. 1), the interplanar distance ${d}_{{\rm{bulk}}}^{444}$ of the structure towards which the films converge was used for the calculation of *K*_σ_. For the 50 nm thick film, *K*_σ_ was found to be 8.6 ± 0.7 kJ/m^3^. The value is close to *K*_u_ confirming the small magnetocrystalline contribution. As expected from the structural relaxation, *K*_σ_ strongly decreases with thickness reaching the smallest value of 0.6 ± 0.7 kJ/m^3^ for the 300 nm thick film. It should be noted that the strain values, which were extracted from the XRD data, are averaged film values. However, it is expected that the relaxation develops gradually along the growth direction rather than the films exhibiting a uniform strain profile, thus leading in turn to thickness-dependent magnetic anisotropies.

Magnetic force microscopy (MFM) was used to image the domain structure of the 70 nm thick film after demagnetization. A typical domain pattern with up and down magnetized domains in the size range of about 300 nm is shown in Fig. 2(b), confirming as well the presence of PMA.

In order to confirm the obtained values of the perpendicular magnetic anisotropy of TmIG films, a ferromagnetic resonance study was performed. The analysis of FMR data was done using the Smit-Beljers approach to derive the resonance conditions from the free energy *F*^34–36^:

1 $${(\frac{\omega }{\gamma })}^{2}=\frac{1}{{(M\sin \theta )}^{2}}({F}_{{\rm{\theta }}{\rm{\theta }}}{F}_{{\rm{\phi }}{\rm{\phi }}}-{F}_{{\rm{\theta }}{\rm{\phi }}}^{2}).$$

where

$$F=\frac{1}{2}{\mu }_{0}{M}_{{\rm{S}}}^{2}{\cos }^{2}\theta -{K}_{{\rm{u}}}{\cos }^{2}\theta -{\mu }_{0}MH(\cos \,\theta \,\cos \,{\theta }_{{\rm{H}}}+\,\cos (\phi -{\phi }_{{\rm{H}}})\sin \,\theta \,\sin \,{\theta }_{{\rm{H}}}).$$

Here, the polar (with respect to film normal) and azimuthal in-plane angles of magnetization *M* and applied field *H* are marked as (*θ*, *φ*) and (*θ*_H_, *φ*_H_), respectively. Note that the film normal is along the [111] crystallographic direction. The FMR spectra were recorded for each sample in the frequency range from 1 to 40 GHz for the in-plane and out-of-plane direction of the magnetic field. In addition, the polar and azimuthal angular dependencies of the resonance field were measured as well to extract the perpendicular magnetic anisotropy and to look for any cubic magnetocrystalline anisotropy contribution, respectively. To determine the resonance field *H*_res_ and resonance peak-to-peak linewidth *ΔH*_pp_, the spectra were fitted using a complex Lorentzian function. Equation (1) was solved numerically at each frequency and (*θ*_H_, *φ*_H_) angles in order to fit the evaluated *H*_res_ values, herewith ${\mu }_{0}{M}_{{\rm{S}}}-2{K}_{{\rm{u}}}/{M}_{{\rm{S}}}\equiv {\mu }_{0}{M}_{{\rm{eff}}}$ was used as a fitting parameter. There are special cases for *θ*_H_ = 0° and *θ*_H_ = 90°, where Eq. (1) can be solved analytically leading to the so-called Kittel formulae for resonance conditions (assuming no cubic anisotropy):

$$\frac{\omega }{\gamma }={\mu }_{0}{H}_{res,\perp }-({\mu }_{0}{M}_{{\rm{S}}}-\frac{2{K}_{{\rm{u}}}}{{M}_{{\rm{S}}}}),\,{\theta }_{H}={0}^{^\circ }.$$

$${(\frac{\omega }{\gamma })}^{2}={\mu }_{0}{H}_{res,\parallel }\times ({\mu }_{0}{H}_{res,\parallel }+{\mu }_{0}{M}_{{\rm{S}}}-\frac{2{K}_{{\rm{u}}}}{{M}_{{\rm{S}}}}),\,{\theta }_{H}={90}^{^\circ }.$$

with $\gamma =g{\mu }_{B}/{\hbar }$, where *g* is the Landé factor. Thus, FMR is a direct method to calculate *K*_u_ using the *M*_S_ value from SQUID–VSM measurements^37^.

**Figure 5:**
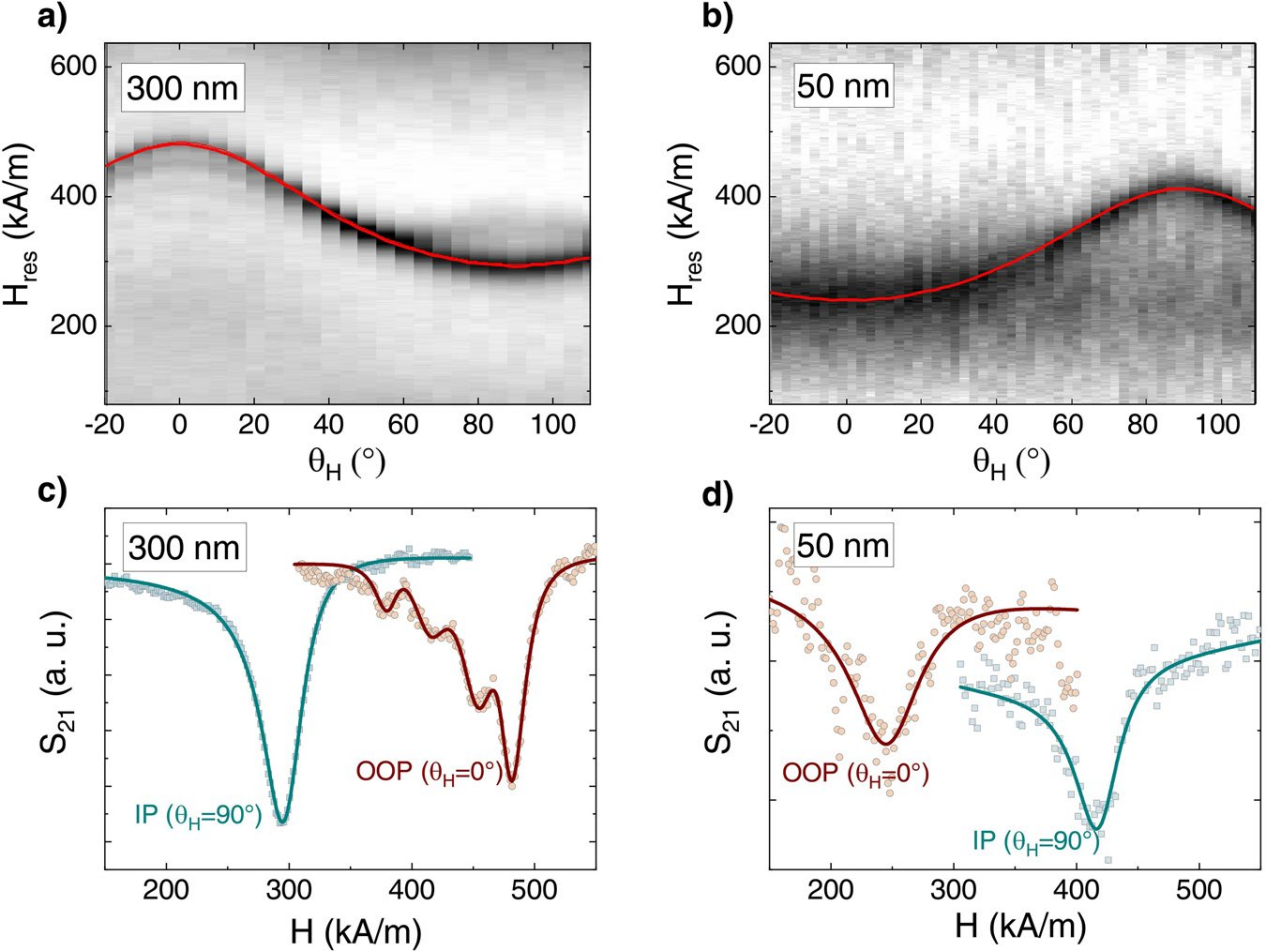
Polar angular dependence of the resonance field *H*_res_ at 10 GHz for TmIG films of (**a**) 300 nm and (**b**) 50 nm thickness, showing an in-plane and out-of-plane easy axis of magnetization, respectively. The red curves are fits according to Eq. (1). Experimental (open symbols) and fitted (solid line) FMR spectra of (**c**) 300 nm and (**d**) 50 nm thick TmIG films, respectively, measured in in-plane (*θ*_H_ = 90°) and out-of-plane (*θ*_H_ = 0°) applied magnetic field. The FMR signal was corrected for background sGGG contribution. For the 300 nm thick film, spin wave modes can be resolved when the magnetic field is applied perpendicular to the film surface.

Figure 5(a,b) shows the detailed polar angular dependencies for TmIG films of 300 nm and 50 nm thickness, demonstrating the opposite behaviour of resonance field with angle *θ*_H_. The field-swept FMR scans are arranged along the y-axis as 2D color plot where the intensity of the FMR signal is marked by color. The individual FMR spectra at *θ*_H_ = 0° and *θ*_H_ = 90° for both films are illustrated by empty symbols in Fig. 5(c,d), while the solid curves represent fits to the experimental data. The background sGGG spectrum was carefully evaluated and subtracted from the presented curves. For the 300 nm thick film, the highest resonance field is observed at *θ*_H_ = 0° which is the direction of the magnetic hard axis. Additionally, in the oop direction (*θ*_H_ = 0°) spin waves up to the 4^th^ mode can be observed on the left side from the main mode. A similar dependence was found for the 200 nm thick TmIG film. In contrast, for the 50 nm thick film the resonance field in *θ*_H_ = 0° direction is the lowest, implying that the easy axis is normal to the film plane. The films of 20 and 70 nm thickness showed similar behaviour. The strong contribution of perpendicular magnetic anisotropy can be clearly concluded for those films. The derived values of *K*_u_ from FMR data analysis are included in Fig. 4(b) by empty red circles. The difference between the *K*_u_ values extracted from FMR and SQUID–VSM data could be explained by the calculating method of *K*_u_ from the hysteresis loops. As mentioned above, *K*_u_ is given by the area enclosed between the oop and ip curves in one quadrant. The strong substrate signal needed to be subtracted can affect the value of the anisotropy field, making the calculation of *K*_u_ values from hysteresis loops error prone. Nevertheless, both techniques give similar values and show clearly the trend of *K*_u_ with film thickness. Please note that the azimuthal (in-plane) angular dependencies of the magnetic resonance field (not shown) did not reveal any magnetocrystalline cubic anisotropy.

The dimensionless Gilbert damping constant *α* was evaluated from the frequency dependence of the resonance peak-to-peak linewidth *ΔH*_pp_ measured in the out-of-plane applied magnetic field for films with thickness 70, 200, and 300 nm, and measured in in-plane applied magnetic field for the two thinnest films (20 and 50 nm). The latter geometry was selected due to the overlapping background signal from the sGGG substrate with the FMR signal when recorded in the easy axis direction which hampered an accurate analysis. The frequency-dependent linewidth was fitted as follows:

$$\Delta {H}_{{\rm{pp}}}=\Delta {H}_{{\rm{inhom}}}+\frac{2}{\sqrt{3}}\frac{\alpha }{\gamma }\omega .$$

where *ΔH*_inhom_ is a constant contribution to the linewidth caused by inhomogeneity of the sample or systematic errors and the second term is the Gilbert damping contribution. The obtained values of the Gilbert damping *α* are shown in Fig. 4(c). The damping constant was found to be in the range of 0.015–0.025, which is comparable with the lowest value of 0.013 so far reported in the literature^25^ still being two orders of magnitude higher compared to YIG, mainly caused by spin-orbit coupling^38^. No clear thickness dependence of the damping constant was observed. The inhomogeneous broadening equivalent to the zero frequency linewidth offset was found to be the lowest for TmIG films with 20 nm (7.16 kA/m ≈ 90 Oe) and 300 nm (5.97 kA/m ≈ 75 Oe) thickness. For intermediate thicknesses of the TmIG films (50, 70, and 200 nm) the value of *ΔH*_inhom_ was as large as 19.90 ± 3.98 kA/m (250 ± 50 Oe) which indicates more pronounced inhomogeneities of the magnetic properties within these films.

## Conclusion

In this study, we have investigated the impact of layer thickness on the structural and magnetic properties of TmIG films grown epitaxially on sGGG(111) substrates. Despite the large lattice mismatch between bulk TmIG and sGGG substrates, films with a thickness up to 70 nm show an oop easy axis of magnetization due to the dominant magnetoelastic anisotropy. With increasing film thickness, we observe a strong relaxation of the induced tensile strain. As a consequence, the direction of the easy axis of magnetization changes gradually throughout the thickness series with a complete rotation towards the film plane for films thicker than 200 nm. Additionally, for all films, a Gilbert damping constant of about 0.02 was obtained, still being two orders of magnitude higher than for YIG films.

## Data Availability

The data that support the findings of this study are available from the corresponding author upon reasonable request.
